# Association between fetal sex and maternal plasma microRNA responses to prenatal alcohol exposure: evidence from a birth outcome-stratified cohort

**DOI:** 10.1186/s13293-020-00327-2

**Published:** 2020-09-10

**Authors:** Nihal A. Salem, Amanda H. Mahnke, Alan B. Wells, Alexander M. Tseng, Lyubov Yevtushok, Natalya Zymak-Zakutnya, Wladimir Wertlecki, Christina D. Chambers, Rajesh C. Miranda, Collaborative Initiative on Fetal Alcohol Spectrum Disorders (CIFASD)

**Affiliations:** 1 Department of Neuroscience and Experimental Therapeutics, College of Medicine, Medical Research and Education Bldg., Texas A&M University Health Science Center, 8447 Riverside Parkway, Bryan, TX 77807-3260 USA; 2 Texas A&M Institute for Neuroscience, Texas A&M University, College Station, TX USA; 3 Women’s Health in Neuroscience Program, Texas A&M University Health Science Center, Bryan, TX USA; 4 Clinical and Translational Research Institute, University of California San Diego, San Diego, CA USA; 5 Department of Pediatrics, University of California San Diego, 9500 Gilman Drive MC 0828, La Jolla, San Diego, CA 92093 USA; 6 Rivne Regional Medical Diagnostic Center, Rivne, Ukraine; 7 Lviv National Medical University, Lviv, Ukraine; 8 OMNI-Net Ukraine Birth Defects Program, Rivne, Ukraine; 9 Khmelnytsky Perinatal Center, Khmelnytsky, Ukraine

**Keywords:** Bootstrap resampling, Sex as a biological variable, Extracellular miRNAs, Fetal alcohol spectrum disorders, Maternal miRNA co-secretion

## Abstract

Most persons with fetal alcohol spectrum disorders (FASDs) remain undiagnosed or are diagnosed in later life. To address the need for earlier diagnosis, we previously assessed miRNAs in the blood plasma of pregnant women who were classified as unexposed to alcohol (UE), heavily exposed with affected infants (HEa), or heavily exposed with apparently unaffected infants (HEua). We reported that maternal miRNAs predicted FASD-related growth and psychomotor deficits in infants. Here, we assessed whether fetal sex influenced alterations in maternal circulating miRNAs following prenatal alcohol exposure (PAE). To overcome the loss of statistical power due to disaggregating maternal samples by fetal sex, we adapted a strategy of iterative bootstrap resampling with replacement to assess the stability of statistical parameter estimates. Bootstrap estimates of parametric and effect size tests identified male and female fetal sex-associated maternal miRNA responses to PAE that were not observed in the aggregated sample. Additionally, we observed, in HEa mothers of female, but not male fetuses, a network of co-secreted miRNAs whose expression was linked to miRNAs encoded on the X-chromosome. Interestingly, the number of significant miRNA correlations for the HEua group mothers with female fetuses was intermediate between HEa and UE mothers at mid-pregnancy, but more similar to UE mothers by the end of pregnancy. Collectively, these data show that fetal sex predicts maternal circulating miRNA adaptations, a critical consideration when adopting maternal miRNAs as diagnostic biomarkers. Moreover, a maternal co-secretion network, predominantly in pregnancies with female fetuses, emerged as an index of risk for adverse birth outcomes due to PAE.

## Introduction

Prenatal alcohol exposure (PAE) is common. For instance, in a recent state-wide study in Texas, we found that 8.4% of assessed newborn infants were positive for a blood alcohol metabolite, indicative of at least 3rd trimester PAE [3]. Moreover, a combination of factors, including high rates of untreated alcohol use disorders [31] and unplanned pregnancies [28], means that PAE is difficult to prevent. Not surprisingly, fetal alcohol spectrum disorders (FASDs), a cluster of physical and neurobehavioral anomalies that can result from PAE [52], are common [35]. In the USA, a recent prospective case ascertainment study showed that between 1.1 and 9% of school-aged children met the diagnostic criteria for FASD [41]. This study also revealed a critical need for better diagnostics, as almost all the identified FASD cases were never previously diagnosed [41]. Moreover, vulnerable groups of children, such as those in state foster care systems, are at significant risk for remaining undiagnosed [1, 18]. The inadequacy of diagnosis results in missed opportunities for early intervention to minimize the effects of PAE and, consequently, increases the risk for secondary scholastic and other disabilities [45].

In attempting to address the critical need for better diagnostic tools, we and others previously reported that maternal plasma miRNAs in both sheep [4] and humans [5, 29] were altered due to PAE. In our study, in a prospective cohort of pregnant women in Ukraine [5], we identified maternal miRNAs that could be used to classify heavy alcohol-exposed pregnancies that resulted in the birth of infants with neurodevelopmental and growth deficits (HEa) as distinct from unexposed pregnancies (UE) or heavy alcohol-exposed pregnancies where the infants were apparently unaffected at birth (HEua). In that study, fetal sex contributed to the accuracy of a machine learning-based prediction model for classifying affected pregnancies [5], raising the intriguing possibility that fetal sex also contributes to the effects of PAE on maternal miRNA profiles.

Sex differences in miRNA secretion emerge as early as the blastocyst stage of embryogenesis and influence maternal endometrial function [32], and sex differences can contribute to maternal physiology into the fetal period [2, 27]. Moreover, fetal sex may also contribute to pregnancy outcomes, including increased risk for pregnancy-related hypertension and preeclampsia [58, 61, 67]. Inherent transcriptomic differences between male and female fetal tissues like the placenta may further drive differences in the fetal response to alterations in the maternal environment, such as maternal exposure to stress (reviewed in [15]). Some studies in animal models and human populations have also documented sex differences in the pathophysiology of FASDs, and suggest that PAE-dependent sex differences can appear during the fetal period itself [42, 49, 51, 64].

Collectively, the above studies support the hypothesis that fetal sex is one determinant of the maternal response to alcohol exposure. Moreover, if maternal plasma miRNAs are to be used as predictive biomarkers for the effects of PAE, the association between fetal sex and alterations in maternal miRNAs needs to be assessed. However, small sample sizes in typical FASD studies in human populations are a significant barrier since the disaggregation of data by fetal sex results in decreased sample size and, therefore, decreased statistical power. To overcome this limitation, we employed a statistical strategy of iterative bootstrap resampling with replacement [11] to simulate characteristics of the population of PAE-exposed pregnancies disaggregated by fetal sex. This methodology has recently been applied to successfully estimate measurement uncertainty in blood tests [21] and in imaging data [39] and is a useful tool for assessing highly disaggregated data for the purposes of building prediction models [50], but to our knowledge, has not been previously applied to studies on FASDs. We report that this strategy uncovered evidence for a stable association between fetal sex and changes in maternal miRNA profiles following PAE. Moreover, these analyses uncovered potential novel regulatory mechanisms for coordinated miRNA secretion into maternal plasma. These data suggest that fetal sex-associated maternal miRNAs are likely to constitute a highly regulated and perhaps adaptive response to PAE that can be leveraged to strengthen the biomarker potential of maternal miRNAs to predict PAE outcomes.

## Methods

### Sample characteristics

All study protocols were approved by the Institutional Review Boards at the University of California San Diego and Texas A&M University and for data collection at Lviv National Medical University in Ukraine. The study sample was drawn from a larger prospective cohort recruited at perinatal clinics in two regions of Ukraine, in the Rivne and Khmelnytsky provinces, by OMNI-Net teams. Recruitment methods and cohort characteristics have been described elsewhere [5, 17, 20]. The study sample included 93 pregnant women, for whom the plasma that met the purity criteria was available (for sample exclusion criteria, see [5]). These included second- and third-trimester plasma samples from 68 women that were profiled previously [5], as well as 25 third-trimester samples from newly assessed subjects. Samples were classified as UE, HEua, and HEa based on the criteria described in Balaraman et al. [5]. Briefly, HEa met the criteria for moderate to heavy drinking, and the child had at least two characteristic alcohol-related craniofacial features (short palpebral fissures, smooth philtrum, and thin vermilion border of the upper lip), growth deficiency, and/or neurobehavioral impairment; HEua met the criteria for moderate to heavy drinking, and the child had no neurobehavioral impairment and no FASD related features; and UE did not meet the criteria for moderate to heavy drinking. Cohort demographic and other characteristics are outlined in Tables 1 and 2.

**Table 1:** Demographic characteristics of the study sample

	HEa (*N* = 34)	HEua (*N* = 23)	UE (*N* = 36)	Overall (*N* = 93)	*p*
Maternal age at enrollment (years)	27.47 ± 6.42	26.35 ± 6.76	27.03 ± 4.76	27.02 ± 5.88	0.631^a^
Gestational age at enrollment (weeks)	18.29 ± 5.06	19.83 ± 5.14	18.03 ± 5.50	18.58 ± 5.24	0.162^a^
Recruitment site					
Khmelnytsky	20 (58.8%)	6 (26.1%)	13 (36.1%)	39 (41.9%)	0.036*^b^
Rivne	14 (41.2%)	17 (73.9%)	23 (63.9%)	54 (58.1%)	
Maternal marital status					
Married or co-habiting	30 (88.2%)	20 (87.0%)	34 (94.4%)	84 (90.3%)	0.506^b^
Single/separated	4 (11.8%)	3 (13.0%)	2 (5.6%)	9 (9.7%)	
Maternal education level					
Less than high school	4 (11.8%)	1 (4.3%)	0 (0.0%)	5 (5.4%)	0.042*^b^
High school or equivalent	18 (52.9%)	13 (56.5%)	13 (36.1%)	44 (47.3%)	
Some college or higher	12 (35.3%)	9 (39.1%)	23 (63.9%)	44 (47.3%)	
Socioeconomic category (Hollingshead Score)					
8–19	8 (23.5%)	1 (4.3%)	2 (5.6%)	11 (11.8%)	0.13^b^
20–29	4 (11.8%)	5 (21.7%)	6 (16.7%)	15 (16.1%)	
30–39	12 (35.3%)	10 (43.5%)	8 (22.2%)	30 (32.3%)	
40–54	8 (23.5%)	5 (21.7%)	13 (36.1%)	26 (28.0%)	
55–66	2 (5.9%)	2 (8.7%)	7 (19.4%)	11 (11.8%)	
Gravidity					
> 1	16 (47.1%)	12 (52.2%)	20 (55.6%)	48 (51.6%)	0.808^b^
1	18 (52.9%)	11 (47.8%)	16 (44.4%)	45 (48.4%)	
Parity					
> 0	14 (41.2%)	10 (43.5%)	16 (44.4%)	40 (43.0%)	0.964^b^
0	20 (58.8%)	13 (56.5%)	20 (55.6%)	53 (57.0%)	
Pre-pregnancy body mass index	23.07 ± 4.22	24.91 ± 3.97	23.55 ± 3.59	23.70 ± 3.94	0.189^a^
Smoking status					
Current smoker	10 (30.3%)	5 (21.7%)	3 (8.3%)	18 (19.6%)	< 0.001*^b^
Never	9 (27.3%)	6 (26.1%)	31 (86.1%)	46 (50.0%)	
Past smoker (quit after realized pregnancy)	11 (33.3%)	7 (30.4%)	1 (2.8%)	19 (20.7%)	
Past smoker (quit before pregnancy)	3 (9.1%)	5 (21.7%)	1 (2.8%)	9 (9.8%)	
Cigarettes per day during pregnancy in smokers	7.10 ± 4.01	4.00 ± 3.16	6.33 ± 3.21	6.11 ± 3.74	0.732^a^
Multivitamin usage after enrollment					
No	12 (35.3%)	3 (13.0%)	8 (22.2%)	23 (24.7%)	0.165^b^
Yes	22 (64.7%)	20 (87.0%)	28 (77.8%)	70 (75.3%)	
Multivitamin usage prior to enrollment					
No	14 (41.2%)	7 (30.4%)	15 (41.7%)	36 (38.7%)	0.687^b^
Yes	20 (58.8%)	16 (69.6%)	21 (58.3%)	57 (61.3%)	
Gestational age (GA) at blood draw					
1st blood draw (GA_bd1_)	19.18 ± 5.09	19.38 ± 5.2	18.21 ± 4.65	18.88 ± 4.93	0.543^a^
2nd blood draw (GA_bd2_)	33.51 ± 2.33	33.57 ± 2.25	32.35 ± 2.50	33.08 ± 2.42	0.013*^a^
AAD: absolute ounces of alcohol per day (*bracketed data in milliliters*)					
AADO: at the time of conception	1.08 ± 1.27 *(31.94 ± 37.56)*	0.63 ± 0.62 *(18.63 ± 18.34)*	0.01 ± 0.06 *(0.3 ± 1.77)*	0.56 ± 0.94 (*16.56 ± 27.8)*	< 0.001*^a^
AADXP: 2 weeks prior to enrollment	0.27 ± 0.65 *(7.98 ± 19.22)*	0.10 ± 0.29 *(2.96 ± 8.58)*	0.00 ± 0.00 *(0.00 ± 0.00)*	0.12 ± 0.43 *(3.55 ± 12.72)*	< 0.001*^a^
AADD: absolute ounces of alcohol per drinking day (*bracketed data in milliliters*)					
AADDO: at the time of conception	2.57 ± 2.53 *(76 ± 74.82)*	1.63 ± 1.44 *(48 ± 42.59)*	0.05 ± 0.21 *(1.48 ± 6.21)*	1.36 ± 2.01 *(40.22 ± 59.44)*	< 0.001*^a^
AADDXP: 2 weeks prior to enrollment	0.80 ± 1.46 *(23.66 ± 43.18)*	0.41 ± 0.74 *(12.13 ± 21.88)*	0.00 ± 0.00 *(0.00 ± 0.00)*	0.39 ± 1.01 *(11.53 ± 29.87)*	< 0.001*^a^
Child sex					
Females	18 (52.9%)	14 (60.9%)	14 (38.9%)	46 (49.5%)	0.246^b^
Males	16 (47.1%)	9 (39.1%)	22 (61.1%)	47 (50.5%)	
Birthweight (g)	^#^3151.85 ± 604.25	3386.09 ± 538.34	3476.50 ± 405.10	3335.45 ± 532.52	0.032*

Demographic characteristics of the study sample. *x* ± *y* represents the mean ± standard deviation

*p values that are statistically significant

^a^Kruskal-Wallis rank sum test

^b^Fisher’s exact test

^#^One mother in the HEa group gave birth to twins, and their birthweight data were eliminated from the calculation of the average birthweight for that group

**Table 2:** Distribution of maternal samples in each trimester by exposure group and infant sex

	UE	HEua	HEa
*Second trimester*			
Total	**25**	**19**	**24**
Males	17	7	11
Females	8	12	13
*Third trimester*			
Total	**36**	**23**	**34**
Males	22	9	16
Females	14	14	18

### Selection of miRNAs for analyses

Plasma samples were processed and assessed for 752 miRNAs using the Exiqon/Qiagen Human panel I+II, V5, miRCURY LNA miRNA miRNome PCR Panel, as described previously, including the assessment of absorbance at 414 nm and mRNA expression of Slc4a1/Band3 to detect hemolysis [5]. We selected miRNAs which were expressed in at least 80% of the samples in all the exposure groups collectively, 153 miRNAs met this expression criteria. For these 153 miRNAs, samples with undetected levels of a miRNA were assigned a cycle threshold (Ct) value of “45” as we previously reported [5]. We then normalized the Ct of each expressed miRNA to the average Ct of all expressed miRNAs in that sample, a normalization strategy that has been shown, in large-scale miRNA expression profiling, to outperform alternate normalization strategies utilizing a set of miRNAs or other small RNA controls [43].

### Statistical analyses

All statistical analyses were implemented in “R” (V3.6.1, R Foundation for Statistical Computing, Vienna, Austria). All “R” code that was developed or adapted for use in these analyses has been deposited at GitHub (https://github.com/nihalasalem/Ukraine_Analysis). For each of the 153 miRNAs, maternal samples for each exposure group (HEa, HEua, and UE) were resampled with replacement for 2000 iterations, consistent with published recommendations [22, 66], and our simulations of statistical parameter stability using an iteration range from 50 to 3000 iterations (Additional file 1). The number of subjects in each resampled group was equal to the number of subjects in each of the HEa, HEua, and UE exposure groups. In each iteration, we reported the results of a statistical test, either ANOVA or ANCOVA *p* values (R Base Package), Hedges’ *g* effect size (effsize R Package), or Pearson’s correlation coefficient (corrplot R package). We then repeated these bootstrap resampling procedures by infant sex, e.g., female HEa, HEua, and UE pregnancies. In each case, the distribution of estimates resulting from iterative resampling is hypothesized to approximate the distribution of the population [26].

#### Analysis of variance

**Fig. 1:**
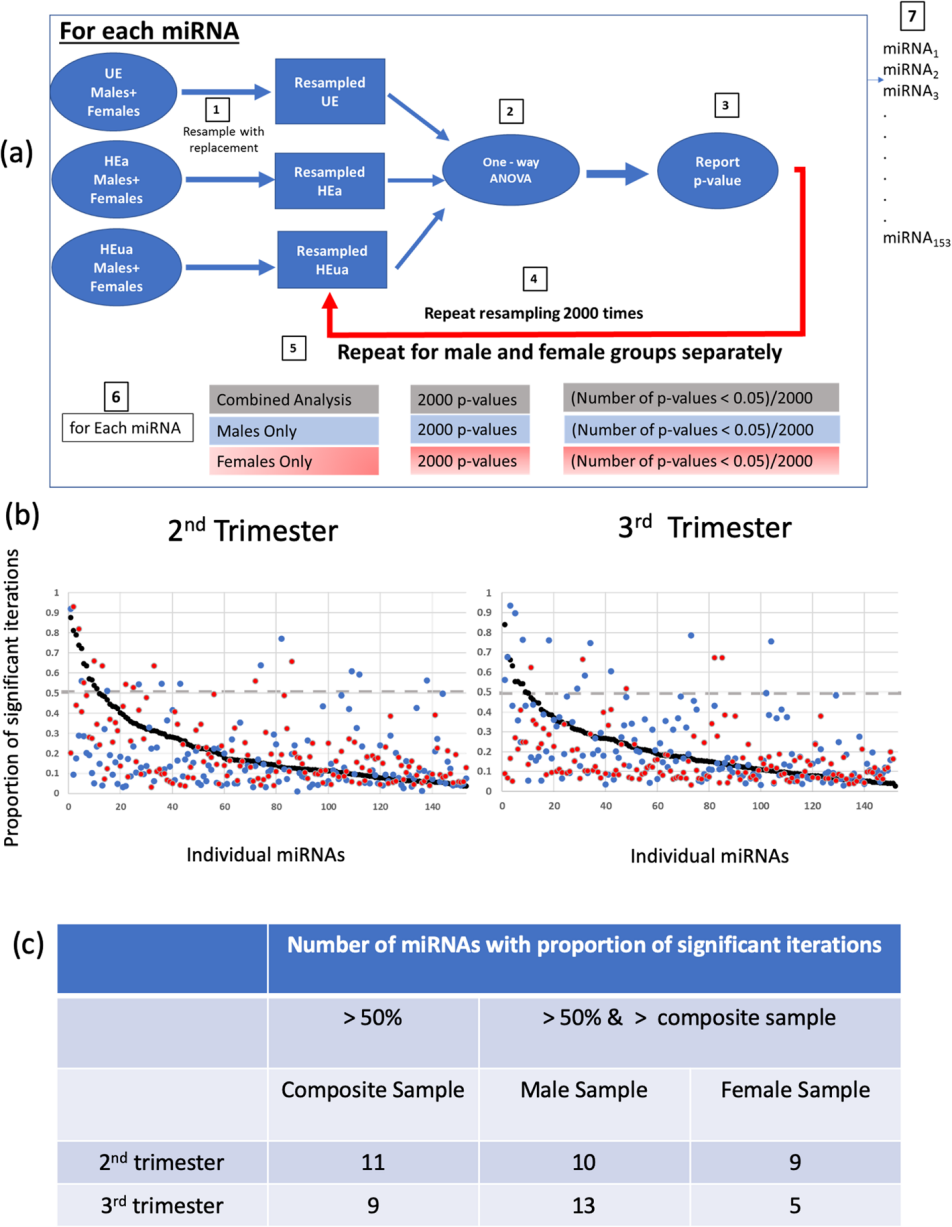
Bootstrap resampling with ANOVA statistical testing identifies sex-specific miRNAs. **a** The schematic workflow for iterative bootstrap resampling with replacement, with the ANOVA as a sample parametric test. (1) Aggregated data in each group were resampled with replacement to generate a new sample with *n* number of observations, identical to the sample size of the original group. (2) A one-way ANOVA was conducted on the three resampled groups. (3) A *p* value from that iteration was recorded. (4) The resampling process was then repeated for 2000 iterations. (5) Steps 1–4 were then repeated for the male and female groups separately. (6) For each miRNA, we report the proportion of iterations in which the ANOVA *p* < 0.05. (7) Steps 1–6 are repeated for each of the miRNAs. **b** The proportion of bootstrap iterations in which a one-way ANOVA was significant (*p* < 0.05) for each miRNA during the second trimester and third trimester. Based on the analyses of the aggregated data (males and females combined, black dots), the miRNAs were ranked from those most likely to be significant in bootstrap reanalysis to those least likely to be significantly altered. Separate resampling was performed for pregnancies with male fetuses (blue dots) and female fetuses (red dots). The gray line indicates the chance probability of reaching statistical significance. **c** Number of “likely alcohol-sensitive, fetal sex-specific” miRNAs in each group

At each time point, 2nd or 3rd trimester, we resampled each exposure group (both infant sexes combined) and conducted a one-way ANOVA on each of the miRNAs in the resampled groups. We reported the *p* value for each miRNA and repeated the resampling process for 2000 times (Fig. 1a). For each miRNA, we calculated the proportion of the iterations in which the ANOVA resulted in a significant *p* value (< 0.05). In a parallel analysis, we re-analyzed the data with bootstrap resampling of an ANCOVA model, with variables, gestational age of blood draw at mid-pregnancy (GA_bd1_) and late pregnancy (GA_bd2_) as covariates, to assess the contribution of the gestational age at blood draw (Table 1) to the stability of parametric estimates derived from the ANOVA model since GA_bd2_ was significantly different across the groups. We then repeated the ANOVA analyses, segregating pregnant mothers into pregnancies that resulted in the birth of male infants or female infants. We then identified miRNAs which exhibited an increased proportion of significant iterations when pregnancies were segregated by infant sex, compared to non-sex-segregated analysis, and labeled these as “likely alcohol-sensitive, fetal sex-specific” maternal miRNAs.

#### Effect size

To investigate the directionality of expression (increased vs. decreased) and group-specific changes of the secreted miRNAs, we performed effect size calculations (with Hedges’ *g* correction for small sample sizes [33]) on the resampled groups comparing each alcohol exposure group to the unexposed group for each of the 153 miRNAs. The median effect size and 95% confidence interval estimates (computed from the 2.5 and 97.5 percentiles of the resulting effect sizes from 2000 bootstrap iterations) resulting from bootstrap resampling analysis were determined for the combined and sex-segregated groups.

### Correlation analyses

#### Primary analyses

We calculated Pearson’s product-moment correlation between each expressed miRNA and all other expressed miRNAs and constructed a correlation matrix for each group (significant correlations, *p* < 0.05). We compared the number of significant correlations between the three exposure groups in the second and third trimester separately. This was followed by a re-analysis where maternal samples were segregated by infant sex. All correlation plots were constructed using the “corrplot” package in “R” [59]. To assess the contribution of the gestational age at blood draw (Table 1) on the correlation coefficients, we calculated the partial correlations (controlling for the gestational age at blood draw variables GA_bd1_ and GA_bd2_) and calculated *R*^2^ between the correlation and the partial correlation coefficients in each exposure group.

#### Bootstrap analyses

To assess potential infant sex-dependent maternal miRNA correlations and control for multiple hypothesis testing [54], we performed bootstrap resampling analyses of Pearson’s correlations. Each exposure group was resampled with replacement for 2000 iterations, where the number of subjects in each resampled group was equal to the number of subjects in each exposure group (HEa, HEua, and UE). In each iteration, we reported the number of significant correlations, and the resulting data from each group were represented in a frequency distribution histogram. We further tested the null hypothesis that the *mean number of significant correlations was not different between the groups*. We counted, in the alcohol-exposed groups (HEa and HEua), the number of significant correlations in each bootstrap iteration which exceeded the mean number of significant correlations in the bootstrap iterations of the non-alcohol exposed groups (UE) and divided this count by the number of bootstrap iterations. The resulted ratio was subtracted from 1 to compute the *p* value. If the *p* value was less than 0.05, the null hypothesis was rejected.

#### Correlations by chromosomal location

For each exposure group, we identified the chromosomal locations of the pairs of miRNAs that were significantly correlated. We next tabulated the frequency of significant correlations between each pair of chromosomes and then calculated the percent change in the number of significant correlations for each pair of chromosomes between the alcohol-exposed and unexposed groups, to derive a measure of inter-chromosome correlations in maternal miRNA expression patterns.

## Results

### Identifying presumptive alcohol-sensitive fetal sex-specific maternal miRNAs

The sample group of 93 pregnant women included in this study differed significantly on the key variable of alcohol consumption (AAD and AADXP, Table 1). A greater proportion of mothers in the HEa and HEua groups also reported current or past smoking, though among self-identified smokers, there were no statistically significant differences in the number of cigarettes consumed per day. Moreover, while there were overall no statistically significant differences between the groups in socioeconomic status, a higher proportion of UE group women reported at least some college-level education (for details about sample demographics, see Table 1).

To identify maternal miRNAs that likely show a fetal sex-dependent response to PAE, we identified the miRNAs which were classified as significant (ANOVA, *p* < 0.05) in more than 50% of the resampling iterations following bootstrap analyses. Overall, sex segregation resulted in a decreased proportion of significant *p* values for most miRNAs, a predicted outcome due to the loss of statistical power with decreased sample size. However, a fraction of miRNAs, between 4 and 7%, exhibited an increased probability of achieving significance when data were segregated by sex (Fig. 1b). These miRNAs exhibited increased proportion of *p* values that exceeded the significance criterion, compared to the combined analyses, and, moreover, exceeded a probability of 0.5 for achieving significance (Fig. 1c, see Additional file 2 for “likely alcohol-sensitive, fetal sex-specific” miRNAs in each group). To assess the potential effects of gestational age at blood draw at mid- and late pregnancy (variables GA_bd1_ and GA_bd2_, Table 1) on the stability of parameter estimates, we additionally combined ANCOVA (with GA_bd1_ and GA_bd2_ as covariates) with bootstrap resampling analysis. We then compared, for each miRNA, the proportion of iterations that were statistically significant (*p* < 0.05) with ANCOVA vs. with ANOVA. This analysis showed a concordance (*R*^2^s between 0.76 and 0.95, see Additional file 3) between ANOVA bootstrap- and ANCOVA bootstrap-derived estimates of parameter stability indicating that GA_bd1_ and GA_bd2_ did not significantly influence outcomes

**Fig. 2:**
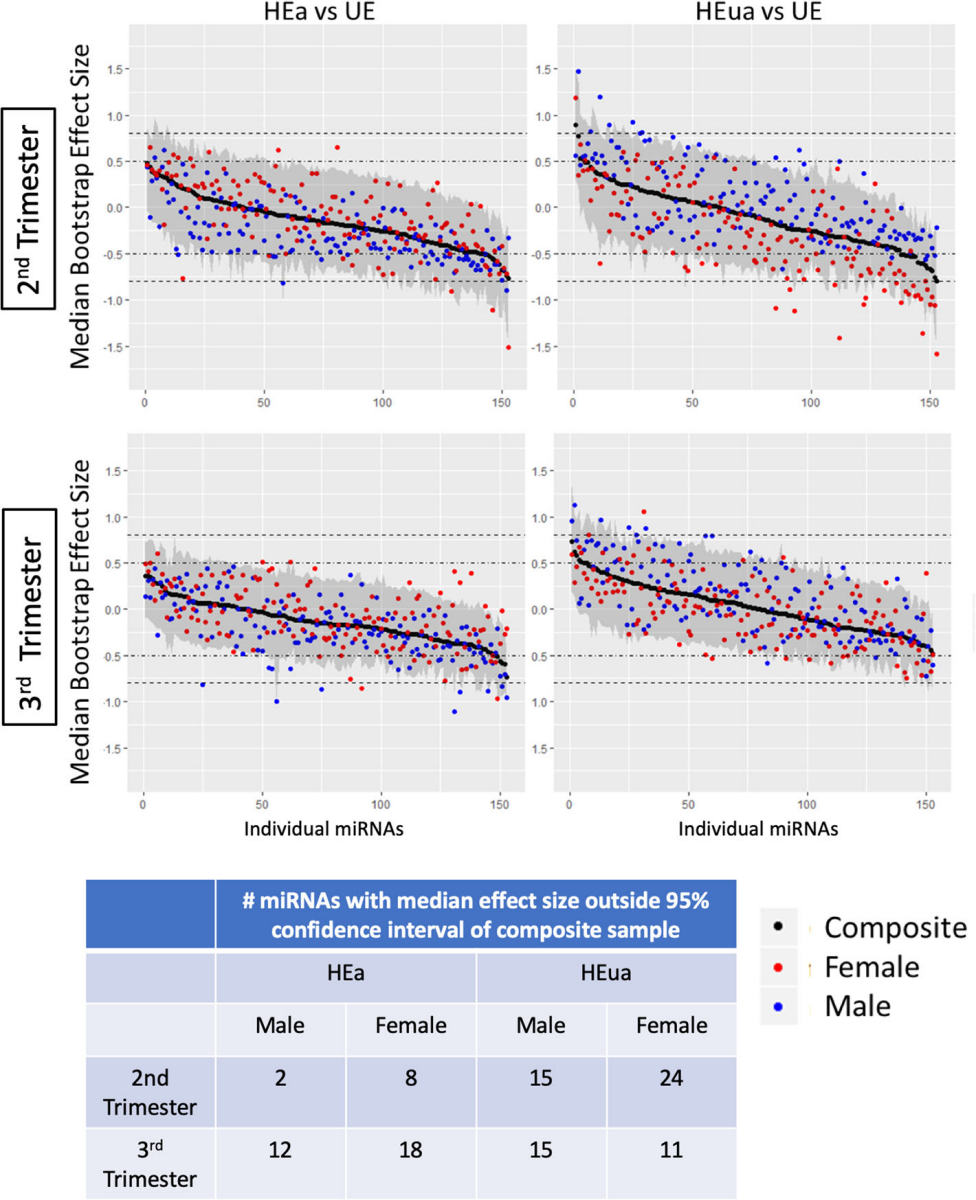
Bootstrap resampling to assess the stability of Hedges’ *g* effect size estimates. Median (black dots) and 95% confidence intervals (gray shading) of the bootstrapped effect sizes for each miRNA for the total sample at the second and third trimester time points. Separate bootstrap analysis of the median effect size of miRNA expression from pregnancies with female (red dots) or male (blue dots) fetuses is overlaid. miRNAs are arranged on the *x*-axis in the order of decreasing median bootstrap effect size in the composite analyses. Dotted lines indicate moderate (0.5) and large (0.8) effect size cutoffs. The table shows the number of miRNAs with sex-segregated median effect size falling outside the 95% confidence interval for the composite sample

**Fig. 3:**
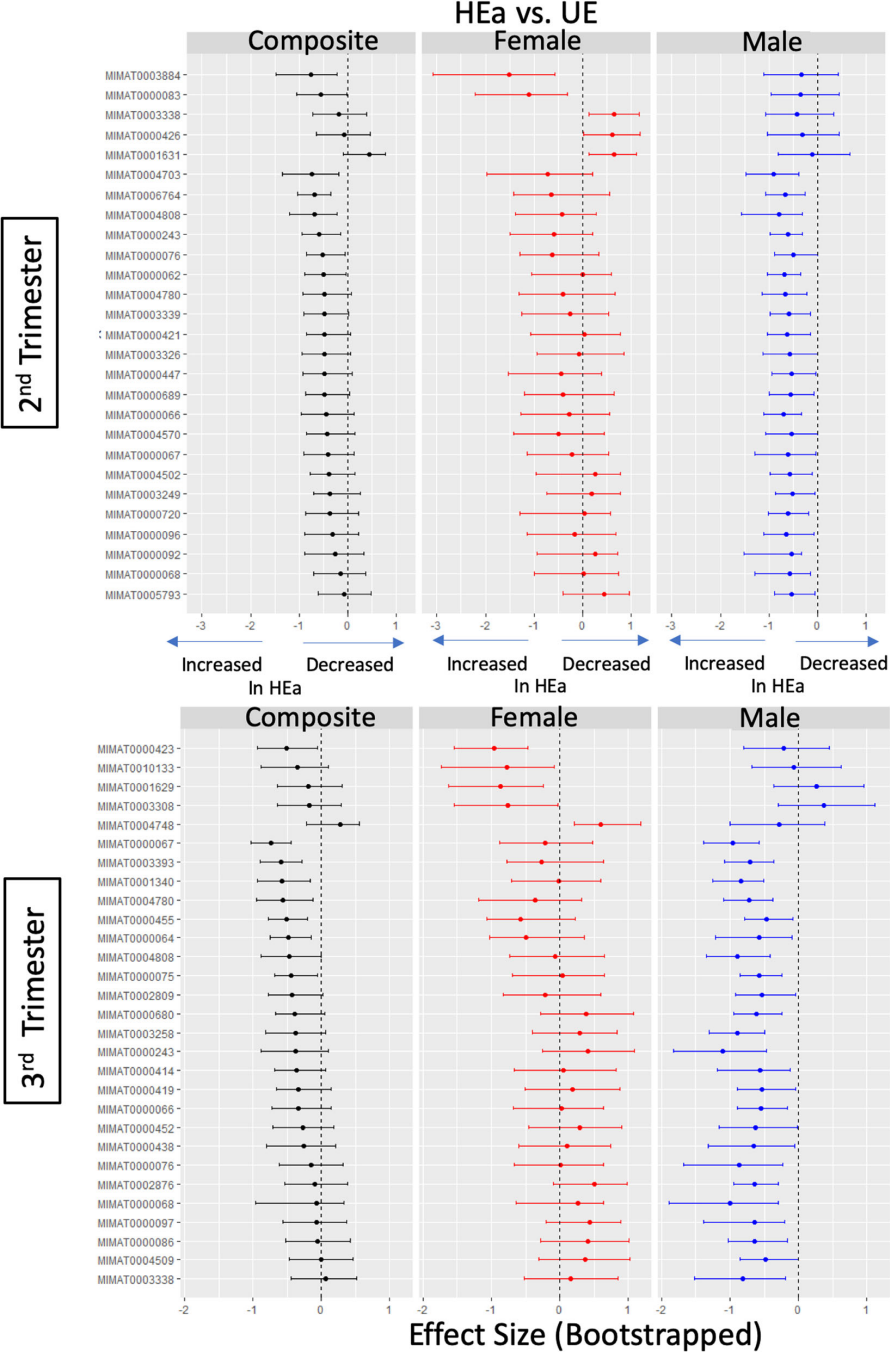
Bootstrap resampling to determine the confidence interval and effect size estimates of the effects of fetal sex. Median bootstrap effect sizes (solid dot) and 95% confidence interval (error bars) for miRNAs which have a non-zero containing confidence interval during sex-segregated resampling. Second and third trimester HEa vs. UE estimates are shown. The miRNA identities of MIMAT numbers (unique accession numbers assigned by miRBase.org) represented in this figure are in Additional file 5. The dashed line indicates zero effect size. The 95% confidence intervals for the resampling distributions for each miRNA were estimated through computing the 2.5 and 97.5 quantiles of the bootstrapped values

To assess the specific contribution of infant PAE outcomes to maternal miRNAs, we conducted a post hoc bootstrap resampling analysis, to compare the effect size (Hedges’ *g*) of changes in maternal miRNA expression in the HEa and HEua groups compared to the UE group (HEa vs. UE, HEua vs. UE). We assessed the stability of group differences first in the composite data and second after disaggregating data by infant sex. A number of sex-segregated miRNAs exhibited median effect sizes that were outside the range of the 95% confidence estimate for the composite sample, but the majority of PAE effects on maternal miRNAs were fetal sex-independent (Fig. 2). miRNAs whose 95% confidence estimates did not include zero were classified as exhibiting a “significant effect.” This analysis identified a number of maternal miRNAs in both HEa and HEua groups, where no effect of PAE (relative to the UE group) was observed in the composite sample, but where there was a significant effect of PAE when the samples were disaggregated by sex (Fig. 3, Additional file 4). For the lists of significant “alcohol-sensitive, fetal sex-specific” miRNAs in each group, see Additional file 5.

To determine if likely sex-specific miRNAs following PAE were due to normally occurring developmental sexual dimorphisms (i.e., differences between control group (UE) mothers who gave birth to male infants vs. those who gave birth to female infants), we segregated the UE population by infant sex, iteratively resampled each group, calculated the effect size due to fetal sex, and after 2000 iterations calculated the median effect size and 95% confidence interval. In the second trimester, 7 miRNAs were significantly decreased, while 4 miRNAs were significantly increased in the plasma of UE mothers who gave birth to male infants compared to those who gave birth to female infants. In the third trimester, 7 miRNAs were significantly decreased while 3 miRNAs were significantly increased in the plasma of UE mothers who gave birth to male infants compared to those who gave birth to female infants. Sex-specific miRNAs in the control, UE, group, identified in the second trimester, did not overlap with those identified in the third trimester. Overall, the fetal sex-specific miRNAs at baseline contributed minimally (0–25%) to the groups of identified alcohol-sensitive fetal sex-specific maternal miRNAs (Table 3).

**Table 3:** Numbers of significantly altered male and female fetal sex-specific miRNAs in each maternal alcohol exposure group, relative to the UE control group

Trimester	Sex-specific at baseline	Male-specific		Female-specific	
		HEa	HEua	HEa	HEua
2nd	11	22 (2)	4 (0)	5 (2)	20 (2)
3rd	10	24 (5)	9 (2)	5 (0)	8 (2)

Number of alcohol-sensitive fetal sex-specific maternal miRNAs as identified by bootstrap analysis of effect size. Numbers in brackets indicate the number of miRNAs showing sex specificity at baseline

### Ethanol exposure results in increased correlated expression of secreted miRNAs in the second and third trimesters in the HEa group

The factors that control miRNA secretion into biofluids are largely unknown. However, evidence for correlated expression of miRNAs would lend support to a hypothesis that expression, or secretion, of circulating miRNAs are co-regulated by a set of common causal mechanisms. To assess the likelihood that maternal miRNAs are co-regulated and/or co-secreted in response to factors in the maternal environment, such as alcohol, we computed the correlation matrices for all expressed miRNAs in each exposure group at mid- and late gestation.

**Fig. 4:**
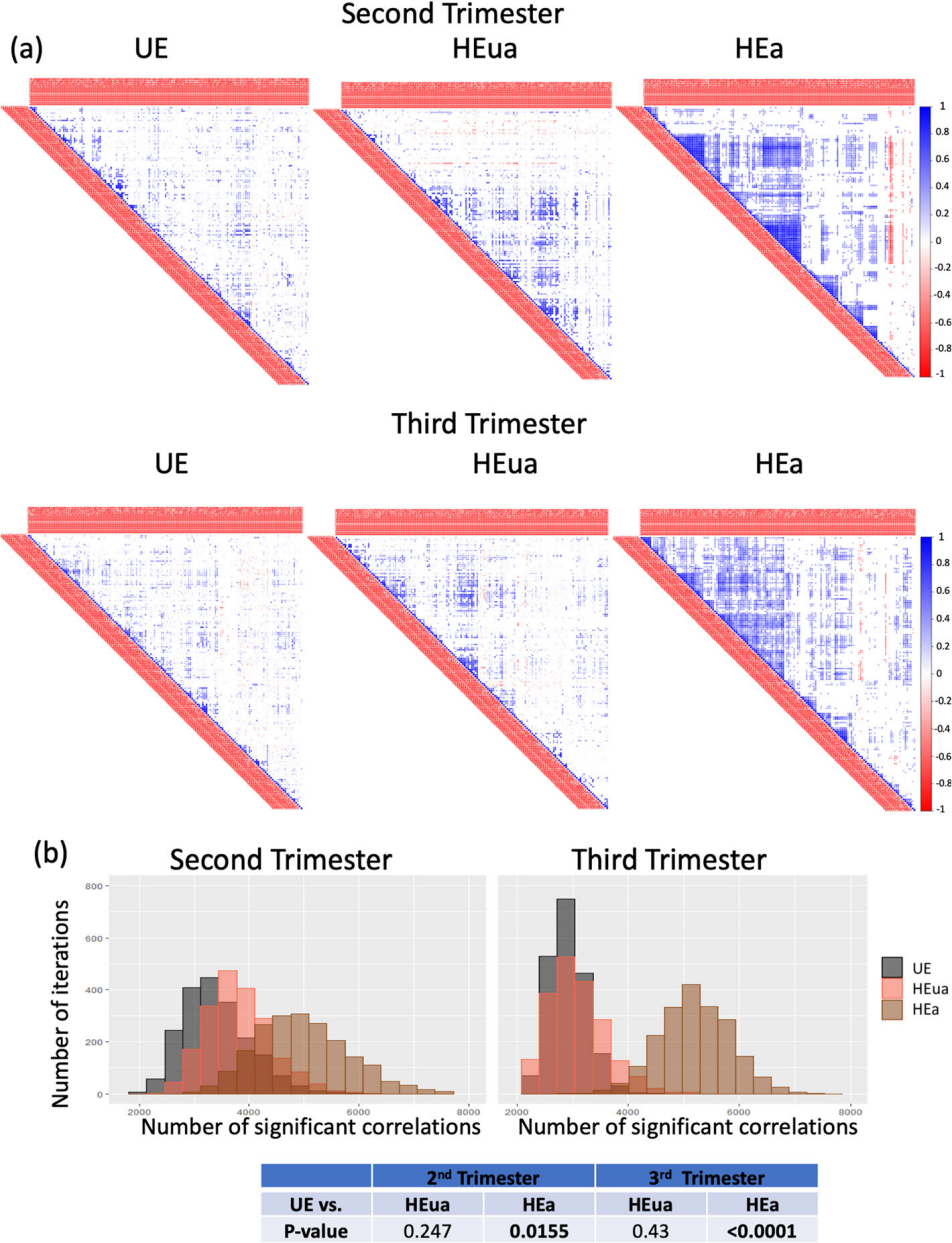
Correlated expression of circulating miRNAs. **a** Correlation plots show significant (*p* < 0.05) cross-correlated expression between miRNA pairs (hierarchically clustered) in each of the exposure groups in the second and third trimesters color-coded by Pearson’s correlation coefficient. **b** Histogram distributions of the number of significant miRNA cross-correlations (*p* < 0.05) in each of the 2000 bootstrap iterations at the second trimester and third trimester time points. The table shows the *p* values for the null hypothesis testing that the mean number of significant correlations in either the HEa or HEua group is not different from the UE group in the second and third trimesters.

PAE increased significant miRNA cross-correlations in the HEa group in both the second and third trimester (Table 4, Fig. 4). To test the stability of those differences in miRNA correlations, we performed bootstrap resampling analyses with replacement within each group and calculated the number of significant correlations in each iteration (data represented as a bootstrap resampling distribution, Fig. 4b). We tested the null hypothesis that the bootstrap resampling-derived mean number of significant correlations was not significantly different across the exposure groups. The bootstrap analyses indicated a statistically significant overall increase in the number of significant correlations in the HEa group compared to the unexposed groups (UE) in the second and third trimesters.

**Table 4:** Number of significant miRNA cross-correlations in each group

	UE	HEua	HEa
*Second trimester*			
**All**	**2421**	**2590**	**3890**
Males	2333	1699	2414
Females	1287	2836	3447
*Third trimester*			
**All**	**2061**	**1935**	**4521**
Males	1804	1492	3168
Females	1864	1792	3760

### Prenatal alcohol’s effect on maternal secreted miRNA cross-correlations is dependent on the fetal sex and birth outcome

We next assessed maternal miRNAs’ cross-correlations segregated by infant sex (Table 4). The numbers of significantly cross-correlated miRNAs in the plasma from mothers who gave birth to male infants were not altered by PAE in the second trimester. In contrast, maternal miRNAs from births that resulted in female infants exhibited an increased number of significant cross-correlations with PAE in the second trimester. However, in the third trimester, infant outcome (whether the infant was born affected) was the main determinant of increased correlations, such that HEa pregnancies had an increased number of significant correlations for both male and female fetuses. To test the possibility that gestational age at blood draw (variables GA_bd1_ and GA_bd2_, Table 1) affected the correlation patterns, we performed partial correlation analysis, controlling for GA, between the maternal miRNAs in each of the exposure groups segregated by infant sex. We observed concordance between correlation and partial correlation estimates (*R*^2^ ranged between 0.856 and 0.995, see Additional file 6) suggesting that the variables GA_bd1_ and GA_bd2_ did not influence patterns of correlation between miRNAs.

**Fig. 5:**
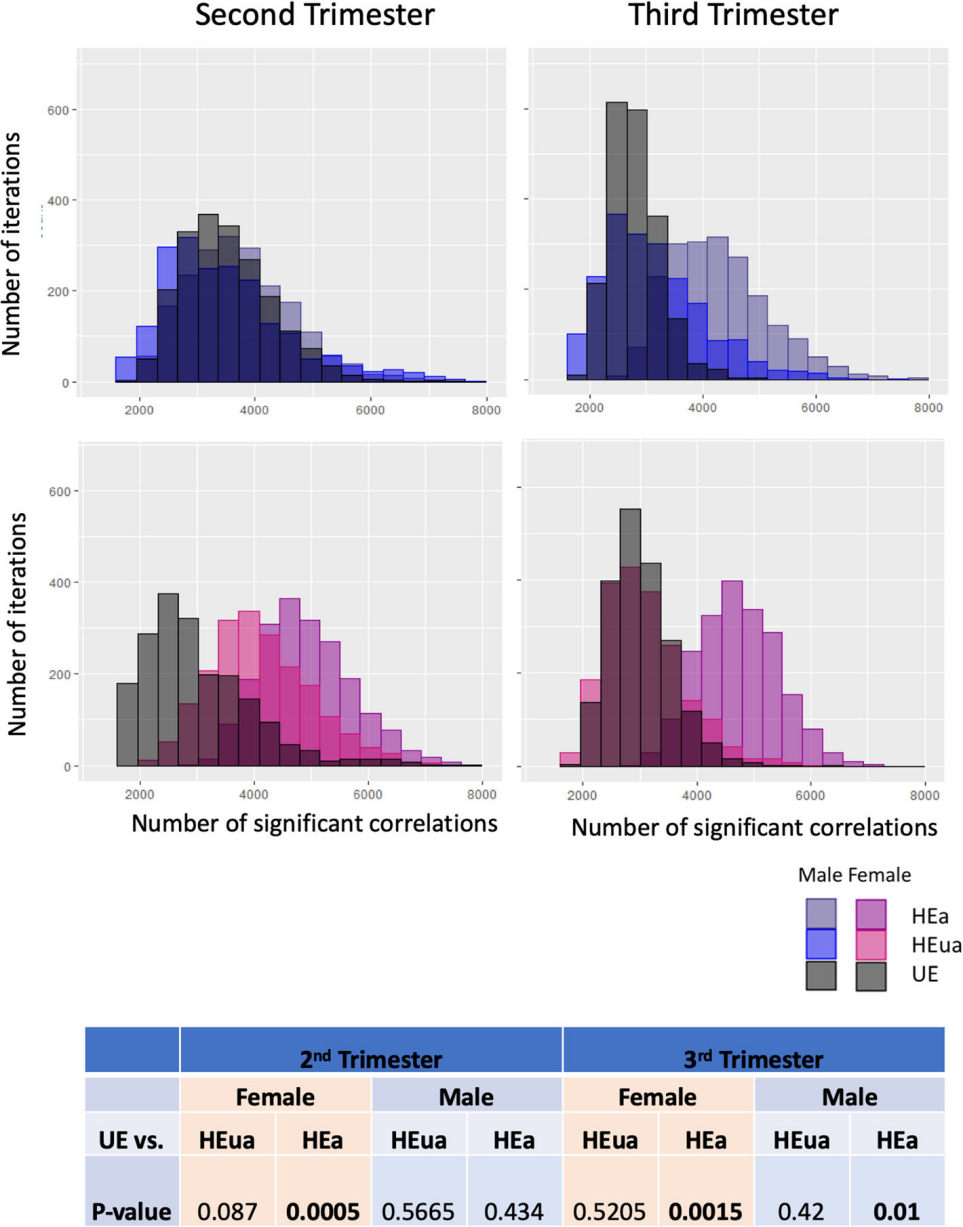
Sex-segregated bootstrap analysis of miRNA expression correlation. Histogram distributions of the number of significant cross-correlations (*p* < 0.05) in each of the 2000 bootstrap iterations at the second trimester and third trimester time points segregated by pregnancies with a male fetus and pregnancies with a female fetus. The table shows the *p* values for the null hypothesis testing that the mean number of significant correlations is not different between each group and the UE group and exposure group for each time point and fetal sex comparison

We used bootstrap resampling analyses to determine the stability of the increased cross-correlations. We tested the null hypothesis that the resampling mean of the number of significant correlations in each of the alcohol-exposed groups (HEa or HEua) was equal to the resampling-mean of the non-alcohol-exposed groups (UE). These data show that in the second trimester, PAE increased the coordinated expression of maternal plasma miRNAs in pregnancies with female fetuses, but not those with male fetuses (Fig. 5). In the 2nd trimester, for mothers of female fetuses, the bootstrap distribution of miRNA cross-correlations in HEua mothers was more similar to the distribution observed with HEa group mothers but shifted to become more similar to UE group mothers by mid-third trimester. Maternal miRNAs in pregnancies with male fetuses exhibited an increased number of significant correlations only in the HEa group, and only in the third trimester, when compared to the UE group.

### Chromosomal location of correlated miRNA expression

**Fig. 6:**
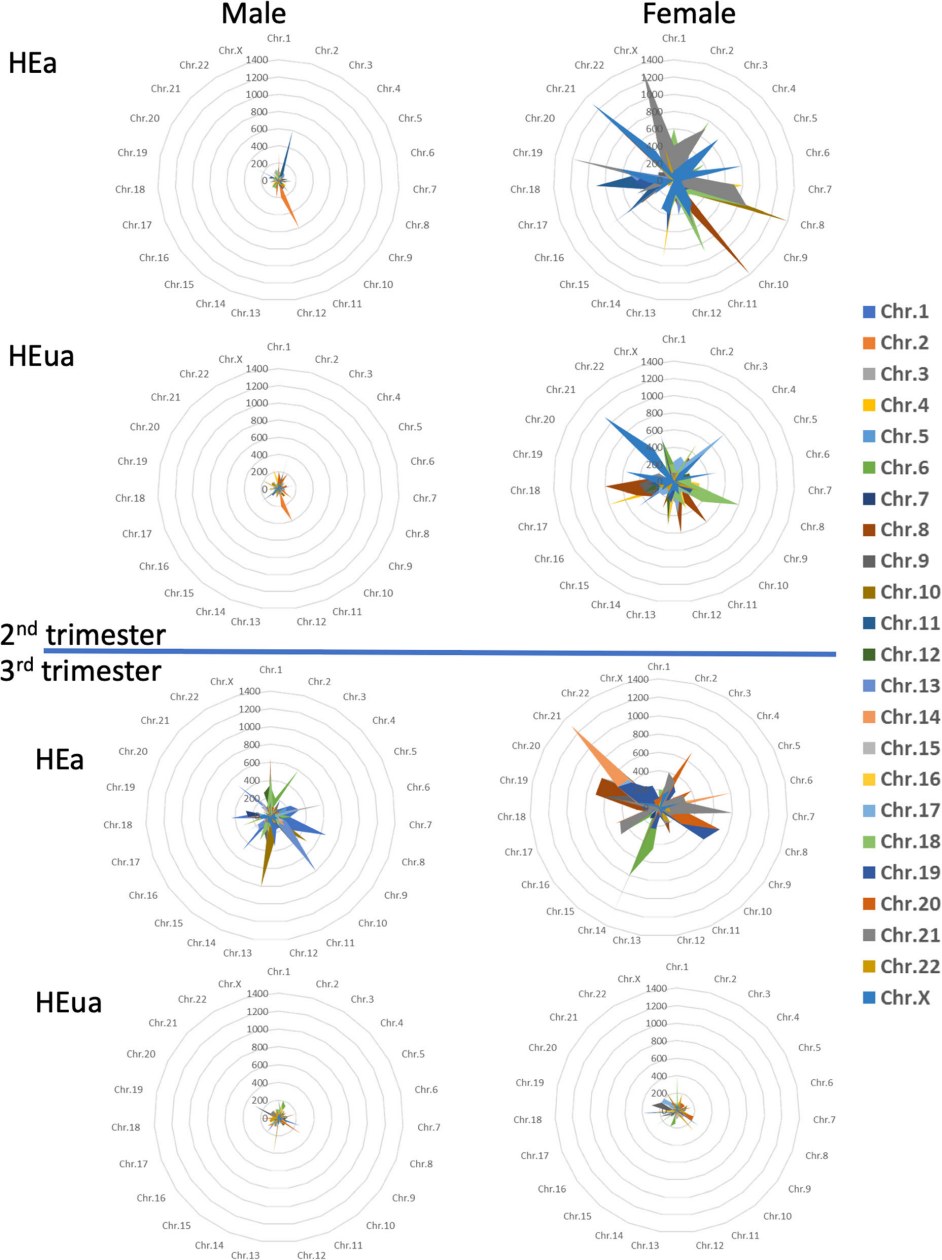
Cross-chromosomal correlations in miRNA expression. Radar plots showing the percent change (HEa or HEua compared to UE) in the number of significant cross-correlations (*p* < 0.05) between miRNAs on each pair of chromosomes at the second- and third trimester time points

The increased number of miRNAs exhibiting correlated expression in pregnancies with female fetuses suggests that either fetal sex chromosomes or fetal endocrine differences (e.g., [48]) may influence maternal plasma miRNA. To assess the potential contribution of chromosomal differences, we examined the cross-correlation of miRNA expression by chromosomal location. We identified the most cross-correlated chromosomes by ranking the percent enrichment of significant correlations between chromosomal pairs in the exposure groups compared to the unexposed groups (e.g., HEua vs. UE and HEa vs. UE). We identified the 10 pairs of chromosomes with the highest enrichment for significant miRNA correlations in each exposure group comparison for pregnancies with fetuses of each sex (chromosomal pairs from each group are listed in Additional file 7). In HEa mothers with female fetuses in the second trimester, X-chromosome-linked miRNAs exhibited a preferential increase in the correlated expression with miRNAs encoded on chromosomes 6 and 21 compared to the UE group mothers. In HEua group mothers with female fetuses, X-chromosome-encoded miRNAs showed an increased correlated expression with miRNAs encoded on chromosomes 4, 6, 19, and 21 compared to UE mothers. In contrast, this enrichment for X-chromosome cross-correlation was not seen in the third trimester in either HEa vs. UE or HEua vs. UE miRNAs from pregnancies with female fetuses (Fig. 6).

## Discussion

The genetic sex of a fetus influences important maternal physiological processes like immune tolerance, inflammation, and activation of the hypothalamic-pituitary-adrenal axis [24, 27, 30, 34, 44, 57]. Fetal sex differences in secreted placental factors may also directly influence placental vasculature and the growth and attachment of the placenta [16], and contribute to the differences in maternal uterine artery blood flow and to maternal vascular adaptations to pathological processes like preeclampsia [14]. Fetal sex can also contribute to the differences in placental function and efficiency in response to maternal disease states like obesity [38] and asthma [46]. Consistent with these observations, previous studies have also identified fetal sex as an important contributory factor to maternal miRNA profiles associated with other gestational pathologies that result in birthweight alterations [53]. However, as is common with small sample size studies, in the above report, none of the identified sex differences survived correction for multiple comparisons, suggesting a need for alternate statistical approaches to disaggregating samples. Here, we utilize an innovative strategy, bootstrap resampling with replacement, as an approach to disaggregating data from small samples, while approximating a population distribution [26], to estimate the association between infant sex and the maternal plasma miRNA response to PAE.

Not surprisingly, ANOVA-bootstrap analyses show that most maternal miRNAs that were PAE-responsive were not influenced by fetal sex. For these miRNAs, when the data were examined in aggregate, statistical significance was observed for up to 90% of the resampling trials. This finding suggests a relatively high degree of homogeneity of samples within each exposure group. However, disaggregating data by fetal sex resulted in a decrease in sample size for each group and, consequently, a loss of statistical power. For these non-fetal sex-dimorphic miRNAs, we observed a reduction in the number of resampling iterations which resulted in statistical significance when data were disaggregated by sex, as would be predicted due to decreased sample size.

Our analyses also show that, for some miRNAs, the genetic sex of the fetus does influence maternal plasma miRNA responses to PAE. For these maternal miRNAs, disaggregating the data by fetal sex resulted in an elevation in the proportion of iterations reaching statistical significance, identifying these maternal miRNAs as “likely fetal sex-specific.” However, a majority of the presumptive fetal sexually dimorphic miRNAs reached statistical significance for less than 80% of the resampling iterations, suggesting the possibility that additional sources of variance aside from fetal sex may contribute to the maternal miRNA profiles. Further analyses are needed to define additional contributory factors that influence both basal and PAE-responsive maternal miRNAs. Subsequent post hoc analyses of effect size also indicated that both HEa and HEua pregnancies exhibited fetal sex-specific miRNAs that were significantly different from UE controls and that these fetal sex-specific patterns were observed in both second and third trimesters. These data emphasize the broader importance of assessing the contribution of fetal sex to maternal biomarkers, as part of the strategy for assay development to predict disease outcomes.

We found that PAE increased the number of significant miRNA cross-correlations, an effect which was modified by both infant sex and birth outcome. In the aggregate data (not accounting for fetal sex), we found that HEa group maternal samples showed an increased number of significant miRNA cross-correlations compared to the HEua and UE groups in both second and third trimesters. Bootstrap resampling analysis, segregating the data by infant sex, showed that the increased number of significant correlations in the HEa group in the second trimester was driven mainly by the presence of a female fetus, whereas in the third trimester, both female fetuses and, to a lesser though still statistically significant extent, male fetuses contributed to significant increases in cross-correlations in the HEa group compared to the UE group. Interestingly, the plasma miRNA cross-correlations in pregnant HEua mothers were intermediate between the HEa group at mid-pregnancy but congruent with the UE group by the end of pregnancy. It is unclear whether there is a causal link between correlated maternal miRNAs and infant birth outcomes. However, this finding raises the intriguing possibility that decreases in the correlated expression of plasma miRNAs may reflect biological mechanisms associated with resiliency, i.e., compensatory biological processes to remediate negative growth and neurobehavioral outcomes from maternal alcohol exposure. Conversely, an increased number of plasma miRNA cross-correlations through the end of pregnancy may be associated with adverse infant birth outcomes.

Research groups have explored the relationships between the expression of miRNAs and their target mRNAs, in the context of alcohol exposures [47, 56, 62] and under basal conditions [37]. However, the correlated expression of miRNAs remains poorly investigated and, when observed, has been hypothesized to be due mainly to the proximity of their encoding locations within chromosomes [7, 19]. Such co-secretion from the miRNA cluster within the14q32 chromosome region has been documented to serve as a biomarker for heart disease [40] and, from the chromosome 19 microRNA cluster, to mediate trophoblast resistance to viral infection [8]. To our knowledge, ours is the first to study to present evidence for correlated expression of miRNAs that are encoded on different chromosomes. For instance, in the 2nd trimester, in HEa and HEua mothers with female fetuses, the expression of X-chromosome encoded miRNAs were significantly correlated with the expression of miRNAs encoded on chromosomes 21 and 6 compared to the UE group.

The correlated behavior of X-chromosome encoded miRNAs was observed specifically in HEa and HEua mothers with female fetuses. However, this finding does not necessarily imply that pregnancies with female fetuses are selectively vulnerable to PAE, since we also observed PAE-linked changes in maternal miRNA profiles in pregnancies with both male and female fetuses following bootstrap-based assessments of effect size. A number of studies have shown that male fetal and postnatal development can also be uniquely sensitive to altered maternal environments due to drug and toxin exposures [6, 23, 25, 55, 65]. However, our analysis does suggest that the toxic effects of PAE may be mediated by different mechanisms in male and female fetuses.

The increased number of miRNA cross-correlations also does not imply that the miRNAs themselves are a causal link between PAE and FASD outcomes. However, the factors that contribute to the correlated miRNA expression may also contribute to the adverse birth outcomes. Firstly, the correlated expression in maternal plasma may be due to the co-transcription of miRNAs in one or a few tissues, such as the placenta, or coordinated secretion of miRNAs from multiple maternal and/or fetal tissues. In the first instance, the increased correlation may be facilitated by the alcohol-driven reorganization of chromatin structure. Chromatin is thought to be structured into topologically associated domains which act as gene regulatory “hotspots” [9], where eukaryotic genes located on separate chromosomes can physically interact [60] and adaptively interact in response to exogenous cues such has hormonal treatments [36], resulting in correlated gene expression changes. Such transcription factories may emerge in a limited set of tissues. For instance, we found evidence in a previous study that a number of miRNAs elevated in maternal circulation in response to alcohol exposure were preferentially transcribed in the placenta [63]. However, correlated patterns of maternal miRNA may also emerge due to stimulated co-secretion from a number of different tissues. Circulating cytokines constitute a candidate class of molecules that may coordinate the secretion of miRNAs into the plasma. For instance, in the same cohort of pregnant women assessed in this study, another group observed that prenatal alcohol exposure also results in elevated levels of circulating cytokines in pregnant women who subsequently gave birth to affected infants [13]. In experimental studies of liver function, cytokines have been shown to induce the release of extracellular miRNAs in exosomes [10]. Consequently, cytokines, perhaps even fetal sex-dimorphic maternal cytokine profiles [27], and extracellular miRNAs may constitute a coordinated signal for future developmental disability due to prenatal alcohol exposure.

While this study does provide preliminary support for the inclusion of fetal sex in predictive models based on extracellular miRNA profiling in mothers, there are several caveats that need to be considered. The assumption behind bootstrap resampling, that the iterative process results in an approximation of the characteristics of a population, may not be generalizable. This method simulates a population to estimate the stability of a statistic, and therefore, the results may not be generalizable to other populations not reflected in the initial sample. Study results may be true for pregnant women from the selected regions in western Ukraine but may not hold true for pregnant women from other geographic locales or from diverse ethnic groups. Variation in the composition of additional risk factors like education, socioeconomic status, nutrition, and poly-substance use may also limit the generalizability of the specific miRNAs identified in the current study population. For instance, in our study population, a higher proportion of HEa and HEua mothers reported smoking during pregnancy compared to UE mothers which is consistent with the evidence for a strong association between tobacco and alcohol consumption behavior [12], though among the smokers in each group, the cigarette consumption levels were similar. The co-use of other psychoactive agents with alcohol may also modify the maternal miRNA profiles. A higher proportion of UE mothers also attained a college education and though not significant overall, UE mothers also tended towards higher socioeconomic status. These factors could be protective and further contribute to changes in maternal miRNA levels. In addition, we classified infants as HEa and HEua based on physical features, growth, and neurodevelopmental assessments through the first year of life. As some alcohol-related neurodevelopmental deficits are not easily detectable until later in childhood, some infants who were truly affected may have been misclassified as unaffected. We would expect this misclassification to result in the attenuation of some of the identified associations. Nevertheless, these limitations do not minimize the need to account for the role of significant variables like fetal sex in the emergence of patterns of biomarkers that predict infant disability.

## Perspectives and significance

This study shows that statistical techniques like bootstrap resampling with replacement can be used successfully, to disaggregate data from small sample FASD studies to generate novel hypotheses. This study opens the door to examining the contributions of other significant maternal health variables to maternal miRNA profiles to make predictive models for the behavior of these biomarkers. These analyses also uncover potentially novel biology of miRNA co-synthesis or co-secretion as an adaptive response to the maternal environment, a phenomenon that predicts diminished fetal resiliency, and needs further investigation. It will also be important to understand why the sex of the fetus results in a change in a maternal miRNA response to PAE and if there is a common sex-dependent behavior in maternal miRNAs responses to other adverse environmental perturbations as well. Our previous studies showed that maternal miRNAs can be biologically active and control the growth of important fetal tissues like the placenta [63]. Consequently, it will be important to ascertain what, if any, biological processes are influenced by such differences. In conclusion, the statistical approaches outlined in this study provide a means of generating hypotheses about the influence of biological variables in studies that were not initially designed to investigate such variables.

## Supplementary information

**Additional file 1.** Selection of the number of bootstrap resampling iterations using second trimester UE group. (a) The frequency distributions of the number of significant miRNA cross-correlations in each of the shown number of iterations. (b) Mean of the number of significant miRNA cross-correlations in each of the shown number of iterations. (c) Mean of the number of significant correlations in each run of 20 simulations using the shown number of resampling iterations.

**Additional file 2.** Likely alcohol-sensitive, fetal sex-specific miRNAs (ANOVA-Bootstrap). Table includes MIMAT numbers, chromosomal location and names of the likely alcohol-sensitive, fetal sex-specific miRNAs for each sex in HEa and HEua in each of the second and third trimesters.

**Additional file 3.** Concordance between ANCOVA-bootstrap & ANOVA bootstrap. X-Y scatterplot showing the relation between the proportion of ANCOVA significant iterations (x-axis) and the proportion of ANOVA significant iterations (y-axis)

**Additional file 4.** Bootstrap-resampling to determine effect size and confidence interval estimates for the effects of fetal sex on maternal HEua miRNA expression. Median bootstrap effect sizes (solid dot) and 95% confidence interval (error bars) for miRNAs which have a non-zero containing confidence interval during sex-segregated resampling. Second and third trimester, HEua vs. UE effect sizes are shown. Dashed line indicates zero effect size.

**Additional file 5.** Significant alcohol-sensitive, fetal sex-specific miRNAs (Effect-Size Bootstrap). Table includes sex-segregated miRNAs that exhibited median effect sizes that were outside the range of the 95% confidence estimate for the composite sample. The table shows the median effect size for each of the composite and sex-segregated analyses.

**Additional file 6. **Concordance between partial correlation analyses correcting for gestational age (GA) at blood draw and full correlation analyses estimates. Figures showing full correlation coefficient between each miRNA pair on X-axis, and partial correlation coefficient correcting for GA on Y-axis, blue line shows the linear regression between the two coefficients. *R*^2^ between the two coefficients are shown. Second trimester groups are shown in (a), third trimester groups are shown in (b).

**Additional file 7.** Table showing the 10 chromosome pairs with the greatest increase in significant cross correlations in either the HEa or HEua groups, relative to the UE group, for pregnancies with male and female fetuses. Percentage of chromosome pair change (%change)= [((no. of significant cross-correlations between miRNAs on a chromosome pair in alcohol exposed group) – (no. of significant cross-correlations between miRNAs on the same chromosome pair in the control, UE, group) * 100)/(no. of significant cross-correlations between miRNAs on the same chromosome pair in the control group)].

## Data Availability

Data on human subjects is deposited at CIFASD.org, in accordance with the NIH data repository guidelines, and is available to outside investigators following an application for data use (https://cifasd.org/data-sharing/). All “R” code that was developed or adapted for use in these analyses has been deposited at GitHub (https://github.com/nihalasalem/Ukraine_Analysis).

## Abbreviations

ANOVA: Analysis of variance
Ct: Cycle threshold
FASDs: Fetal alcohol spectrum disorders
HEa: Heavily exposed with affected infants
HEua: Heavily exposed with apparently unaffected infants
MIMAT: Mature miRNA miRBase sequence entry unique accession number
miRNAs: MicroRNAs
PAE: Prenatal alcohol exposure
UE: Unexposed to alcohol
