# Supplementary figures and images for: Association between fetal sex and maternal plasma microRNA responses to prenatal alcohol exposure: evidence from a birth outcome-stratified cohort
Source: Biol Sex Differ. 2020 Sep 10;11:51. doi: 10.1186/s13293-020-00327-2 (PMC7488011; doi:10.1186/s13293-020-00327-2)

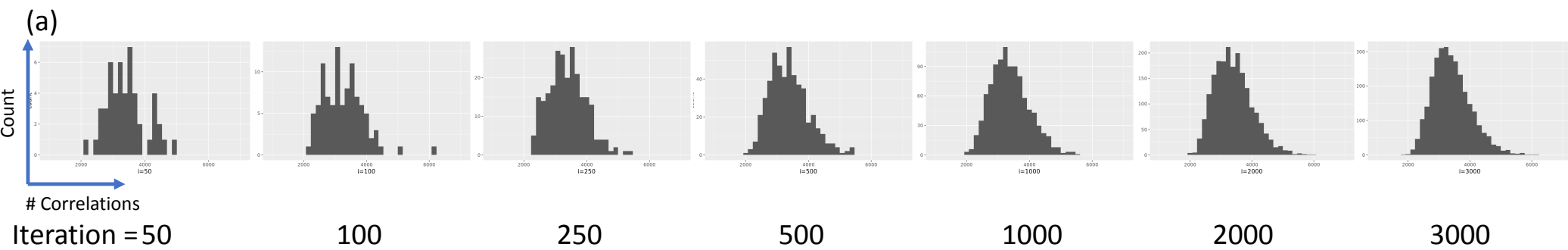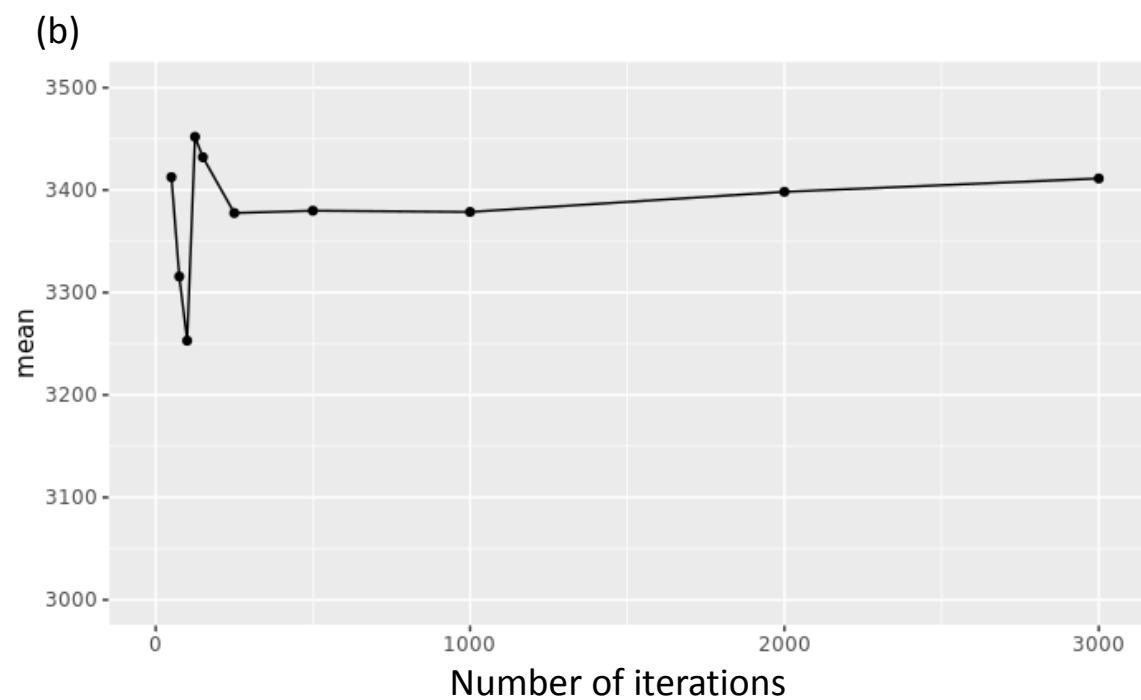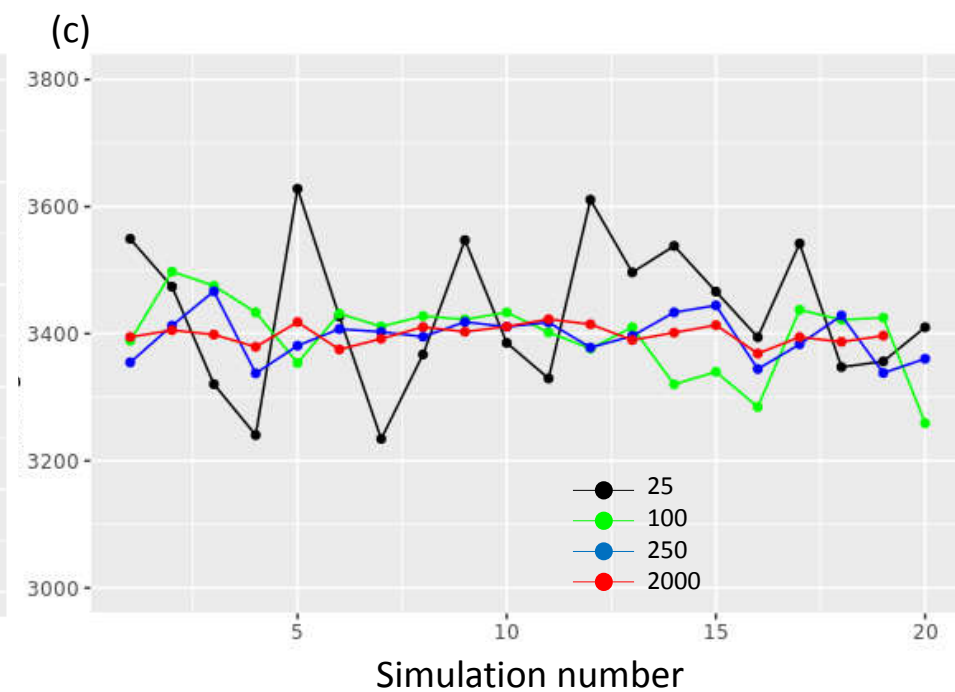

Supplement: Supplementary file 1 — Additional file 1. Selection of the number of bootstrap resampling iterations using second trimester UE group. (a) The frequency distributions of the number of significant miRNA cross-correlations in each of the shown number of iterations. (b) Mean of the number of significant miRNA cross-correlations in each of the shown number of iterations. (c) Mean of the number of significant correlations in each run of 20 simulations using the shown number of resampling iterations. [file 13293_2020_327_MOESM1_ESM.pdf]

2<sup>nd</sup> Trimester

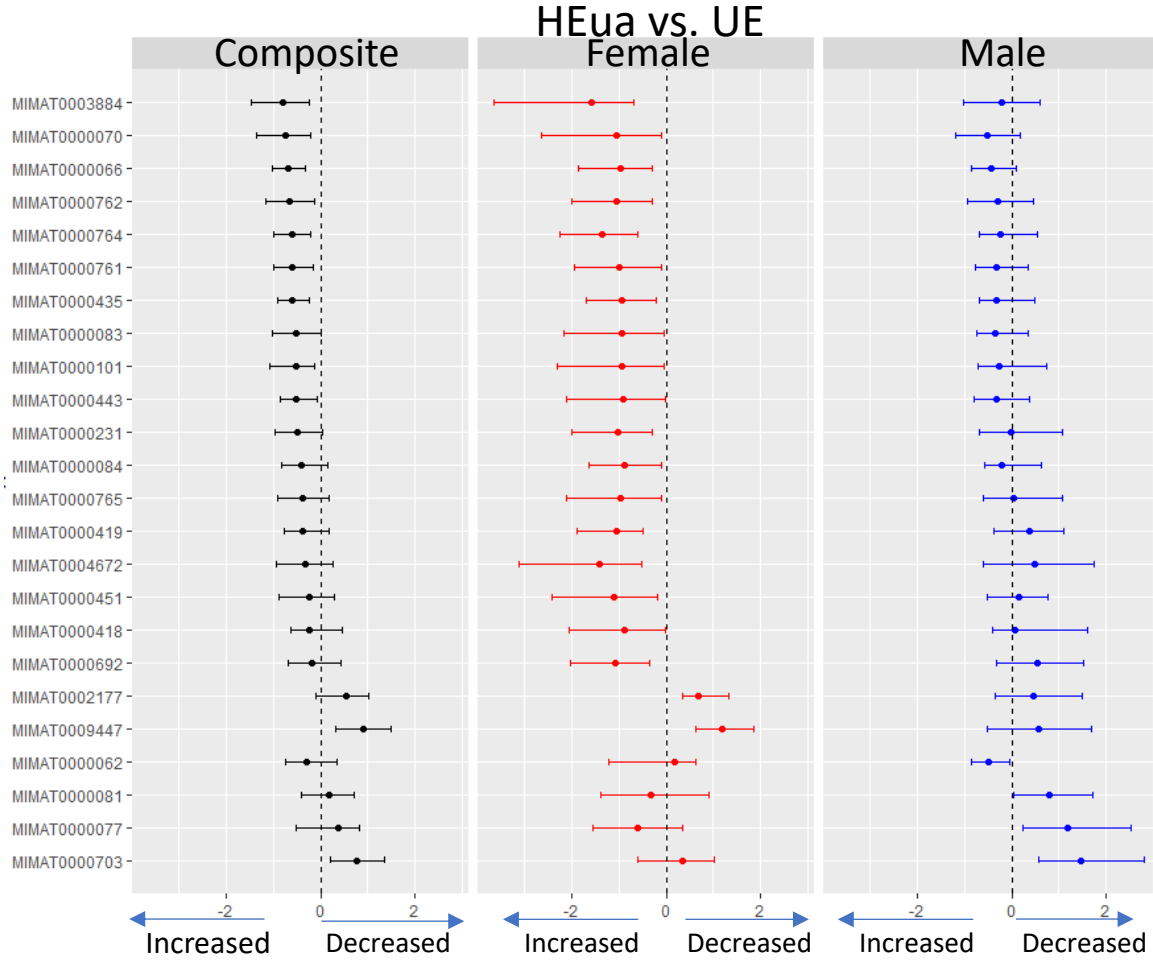

3<sup>rd</sup> Trimester

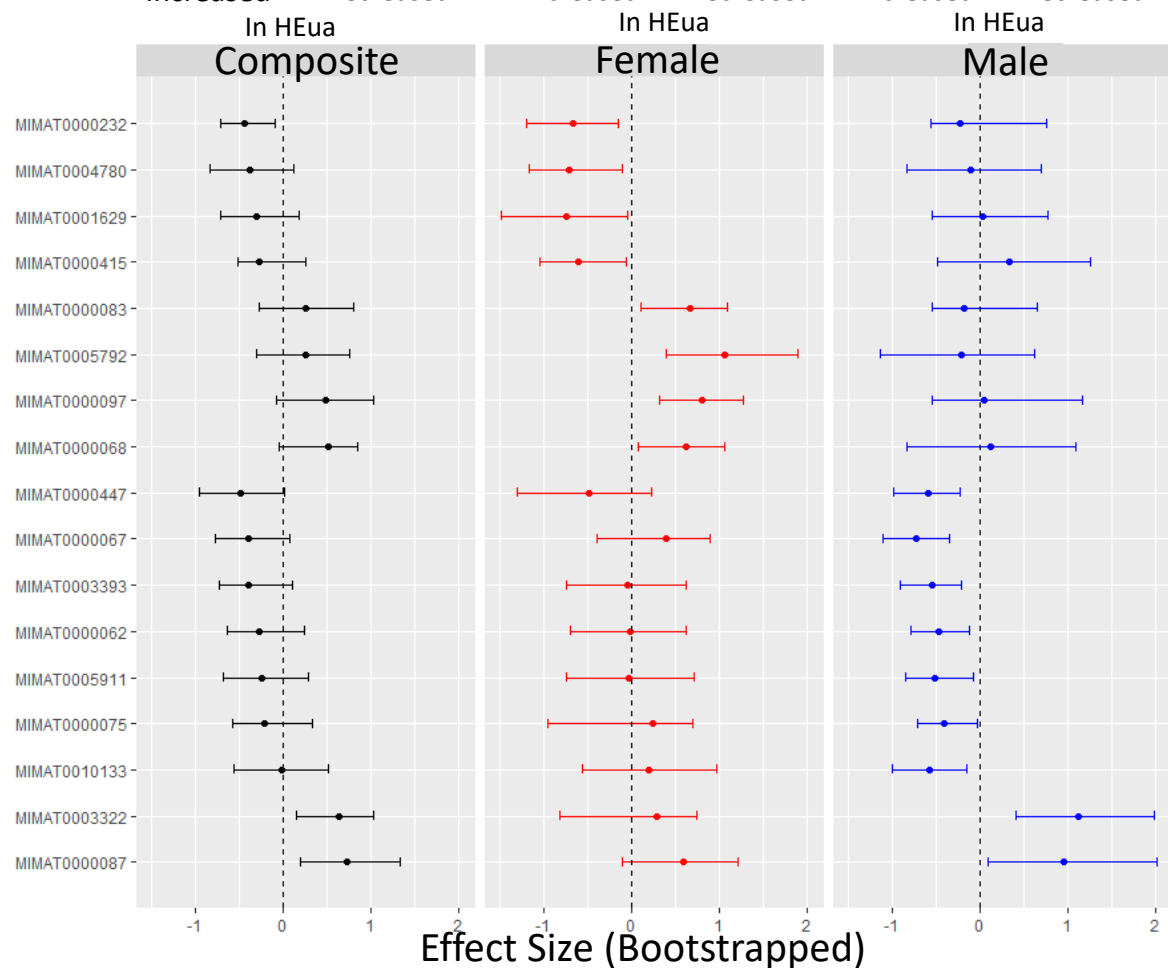

Supplement: Supplementary file 4 — Additional file 4. Bootstrap-resampling to determine effect size and confidence interval estimates for the effects of fetal sex on maternal HEua miRNA expression. Median bootstrap effect sizes (solid dot) and 95% confidence interval (error bars) for miRNAs which have a non-zero containing confidence interval during sex-segregated resampling. Second and third trimester, HEua vs. UE effect sizes are shown. Dashed line indicates zero effect size. [file 13293_2020_327_MOESM4_ESM.pdf]
