# Supplementary material for: Association between fetal sex and maternal plasma microRNA responses to prenatal alcohol exposure: evidence from a birth outcome-stratified cohort
Source: Biol Sex Differ. 2020 Sep 10;11:51. doi: 10.1186/s13293-020-00327-2 (PMC7488011; doi:10.1186/s13293-020-00327-2)
Supplement: Supplementary file 3 — Additional file 3. Concordance between ANCOVA-bootstrap & ANOVA bootstrap. X-Y scatterplot showing the relation between the proportion of ANCOVA significant iterations (x-axis) and the proportion of ANOVA significant iterations (y-axis) [file 13293_2020_327_MOESM3_ESM.pdf]

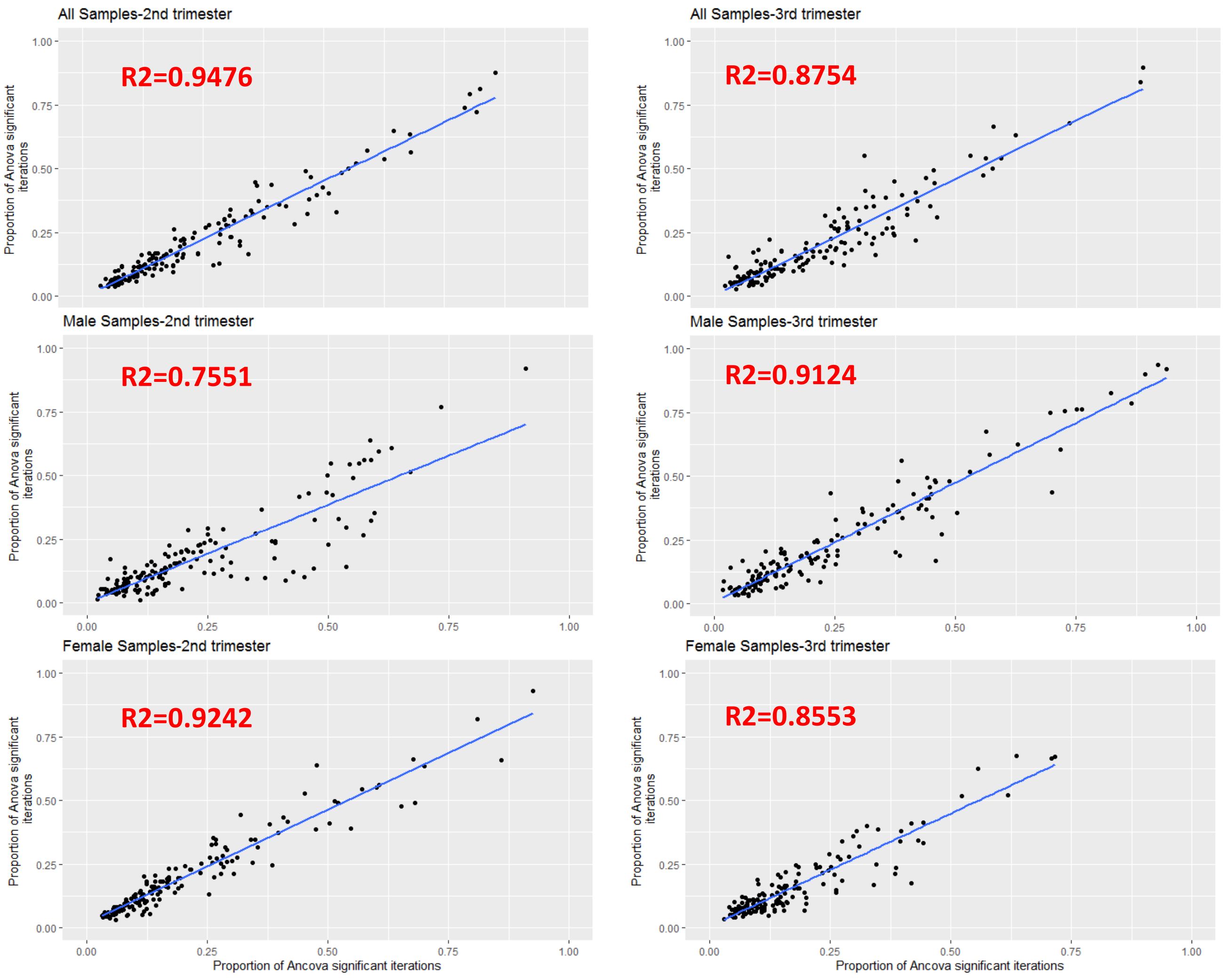
All Samples-2nd trimester
R2=0.9476
All Samples-3rd trimester
R2=0.8754
Male Samples-2nd trimester
R2=0.7551
Male Samples-3rd trimester
R2=0.9124
Female Samples-2nd trimester
R2=0.9242
Female Samples-3rd trimester
R2=0.8553
Proportion of Anova significant iterations
Proportion of Ancova significant iterations
0.00
0.25
0.50
0.75
1.00
