# Supplementary material for: Association between fetal sex and maternal plasma microRNA responses to prenatal alcohol exposure: evidence from a birth outcome-stratified cohort
Source: Biol Sex Differ. 2020 Sep 10;11:51. doi: 10.1186/s13293-020-00327-2 (PMC7488011; doi:10.1186/s13293-020-00327-2)
Supplement: Supplementary file 6 — Additional file 6. Concordance between partial correlation analyses correcting for gestational age (GA) at blood draw and full correlation analyses estimates. Figures showing full correlation coefficient between each miRNA pair on X-axis, and partial correlation coefficient correcting for GA on Y-axis, blue line shows the linear regression between the two coefficients. R2 between the two coefficients are shown. Second trimester groups are shown in (a), third trimester groups are shown in (b). [file 13293_2020_327_MOESM6_ESM.pdf]

(a)

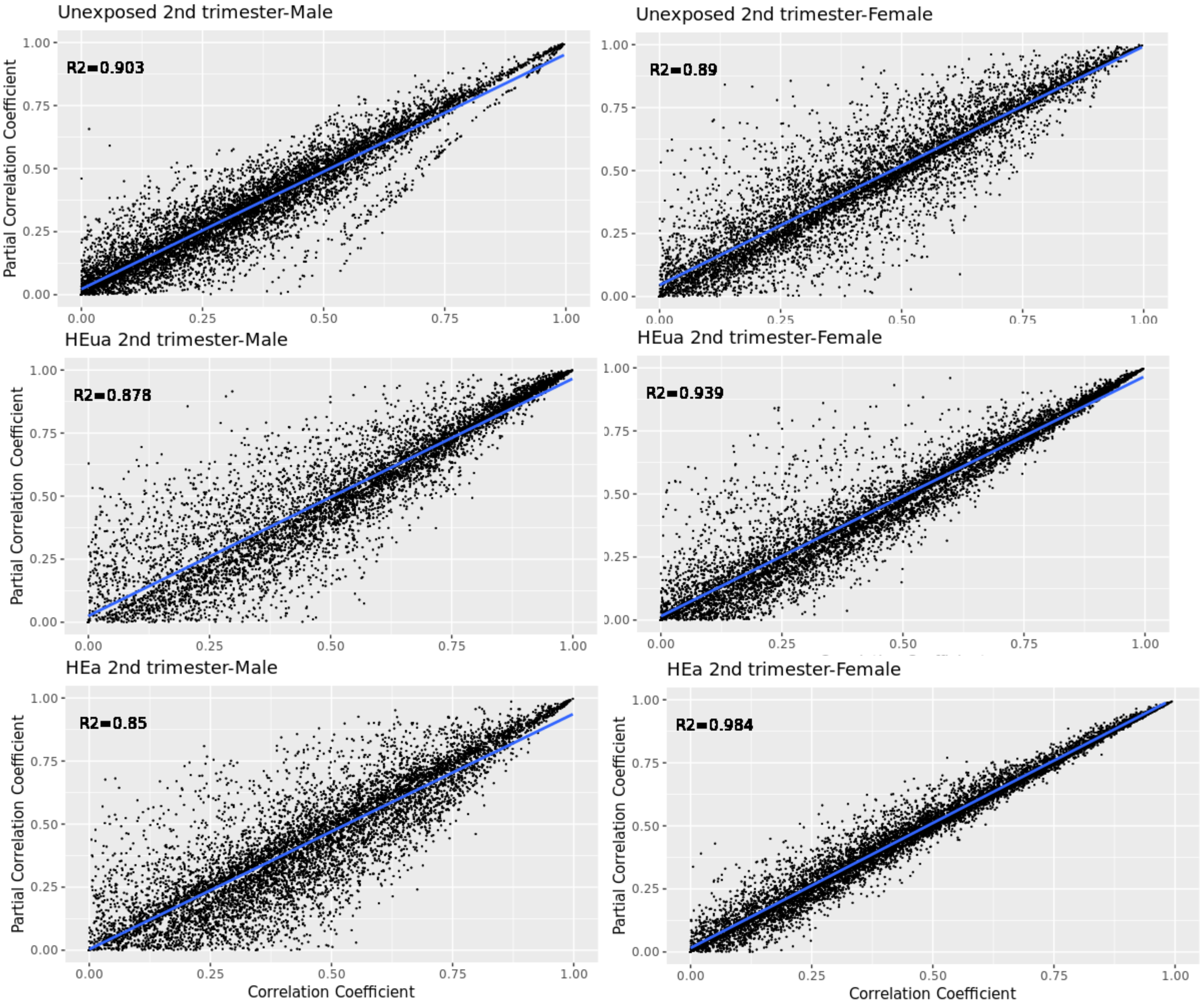

Unexposed 2nd trimester-Male
R2=0.903
Unexposed 2nd trimester-Female
R2=0.89
HEua 2nd trimester-Male
R2=0.878
HEua 2nd trimester-Female
R2=0.939
HEa 2nd trimester-Male
R2=0.85
HEa 2nd trimester-Female
R2=0.984
Partial Correlation Coefficient
Correlation Coefficient
0.00
0.25
0.50
0.75
1.00

(b)

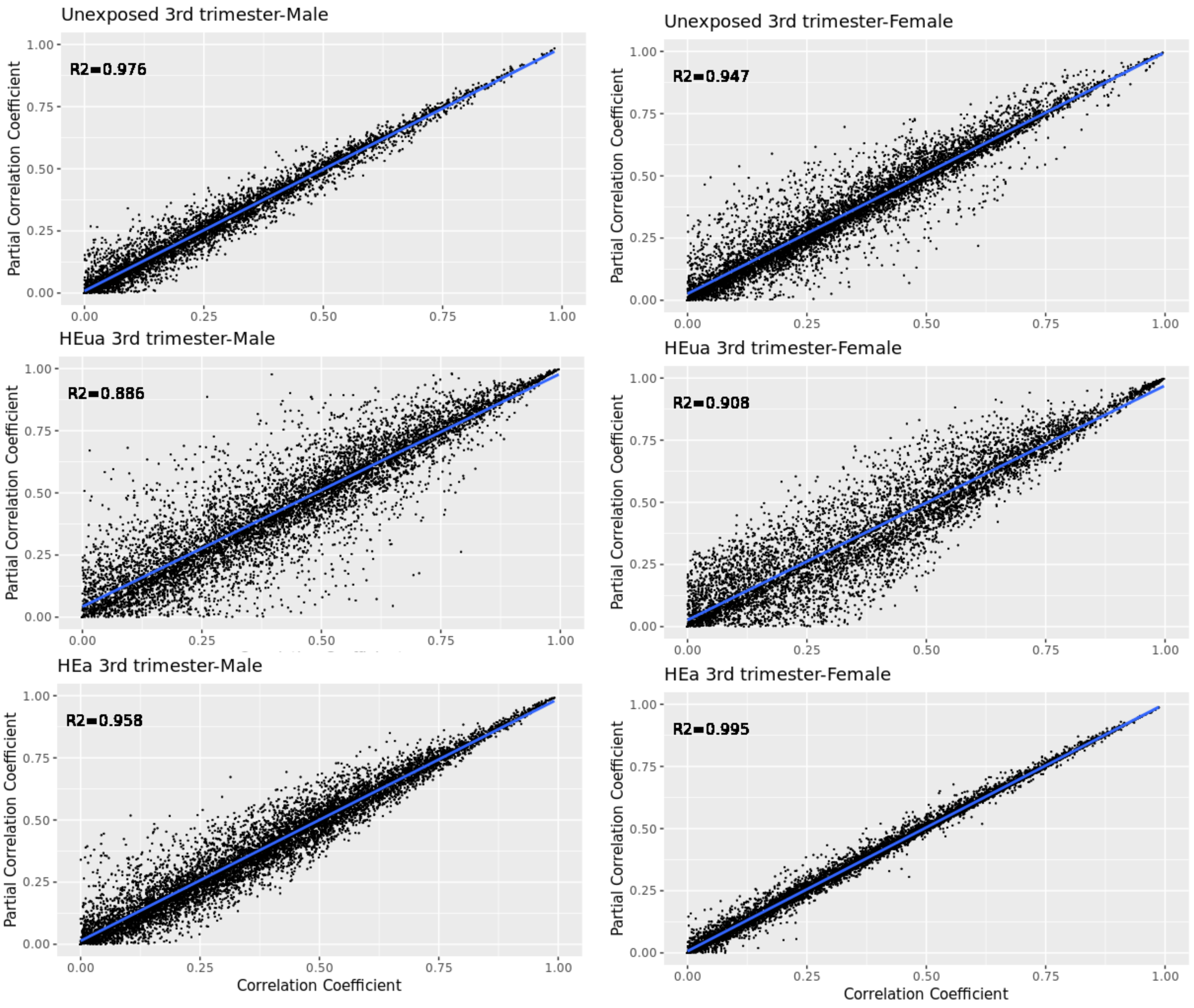

Unexposed 3rd trimester-Male
R2=0.976
Partial Correlation Coefficient
Unexposed 3rd trimester-Female
R2=0.947
HEua 3rd trimester-Male
R2=0.886
HEua 3rd trimester-Female
R2=0.908
HEa 3rd trimester-Male
R2=0.958
HEa 3rd trimester-Female
R2=0.995
Correlation Coefficient
0.00
0.25
0.50
0.75
1.00
